# Chorordin-like 1 inhibits pancreatic cancer cell migration and invasion: involvement of the BMP4/SMAD pathway

**DOI:** 10.3389/fonc.2025.1633464

**Published:** 2025-08-05

**Authors:** Wei Li, Yalan Zhong, Yuqiao Song, Hongmei Wang, Zheng Jiang

**Affiliations:** ^1^ Department of Gastroenterology, The First Affiliated Hospital of Chongqing Medical University, Chongqing, China; ^2^ The Second People’s Hospital of Yubei District, Chongqing, China

**Keywords:** pancreatic cancer, CHRDL1, BMP4/SMAD, migration, invasion

## Abstract

**Introduction:**

Pancreatic cancer is a highly aggressive malignancy with a 6% five-year survival rate. CHRDL1, a BMP4 antagonist, has tumor-suppressive effects in breast and gastric cancers, but its role in pancreatic cancer is unclear. This study explores CHRDL1’s function and mechanism in pancreatic cancer.

**Methods:**

Stably transfected pancreatic cancer cell lines (PANC-1, SW1990) with lentivirus-mediated CHRDL1 overexpression were established to assess effects on cell proliferation, migration, and adhesion. Recombinant BMP4 treatment validated CHRDL1’s antagonism. Additionally, the TCGA database, immunohistochemistry, and RT-qPCR in both cell lines and patient tissues confirmed CHRDL1 expression. In vivo experiments were also conducted to observe the effect of CHRDL1 overexpression on pulmonary metastases.

**Results:**

CHRDL1 was downregulated in pancreatic cancer, correlating with poor prognosis. Overexpression inhibited cell migration and adhesion (without affecting proliferation), reduced SMAD1/5/9 phosphorylation and RUNX2 expression, and counteracted BMP4-induced malignant behaviors.

**Discussion:**

CHRDL1 exerts tumor-suppressive effects in pancreatic cancer by inhibiting the BMP4/SMAD pathway, reducing migration, invasion, and metastasis. These findings clarify CHRDL1’s role, enhance understanding of pancreatic cancer mechanisms, and may offer diagnostic and therapeutic targets.

## 1 Introduction

Pancreatic cancer has the worst prognosis among all cancers, with a 5-year survival rate of only 6% (1). In 2017, there were a total of 441,000 cases of pancreatic cancer globally (2). As the problem of an aging population becomes increasingly significant, the morbidity and mortality of pancreatic cancer rises sharply with age (3). The risk factors of pancreatic cancer include cigarette smoking, obesity, diabetes, and alcohol intake (4). Due to the high metastatic potential and proliferative characteristics of pancreatic cancer cells, up to 80% of patients are diagnosed with advanced tumors that are unresectable or metastatic (5). Currently, gemcitabine-based systemic chemotherapy in combination with nab-paclitaxel or oxaliplatin has been shown to improve long-term survival in advanced pancreatic cancer (6, 7). Therefore, Exploring the pathogenesis of pancreatic cancer is a critical research strategy for treating the disease.

Bone morphogenetic proteins (BMPs) are secreted proteins that belong to the transforming growth factor (TGF)-β family (8). BMP type I and type II receptors, which are serine/threonine kinase receptors, are responsible for transducing BMP signaling (9). BMP ligands bind to type I and II BMP receptors, forming a heterotetrametric complex that phosphorylates the type I receptor by the type II kinase domain and activates SMAD1/5/9 proteins (10). Subsequently, the phosphorylated SMAD1/5/9 proteins form heterodimeric complexes with SMAD4 and are transported into the nucleus, where they interact with transcription factors such as RUNX2, stimulating cellular behaviors like proliferation and migration (11). According to Hamada et al., BMP4 exacerbates pancreatic carcinoma development by promoting epithelial-to-mesenchymal transition, whereas BMP4 downregulation is thought to be protective (12).

BMP expression and activity are regulated by a series of intracellular and extracellular proteins called BMP antagonists, which typically bind to BMP family ligands and prevent them from interacting with receptors, thereby inhibiting signal transduction (13). The delicate balance between BMP and BMP antagonist activity is thought to be a major contributing factor to malignant transformation and progression in the cancer (14). Chordin like-1 (CHRDL1), as a BMP antagonist, is a secreted glycoprotein (15). CHRDL1 exhibits dual regulatory roles in tumorigenesis and progression. As demonstrated by Dong H et al., CHRDL1 significantly improves patient prognosis in lung adenocarcinoma by suppressing TGF-β signaling-mediated epithelial-mesenchymal transition (EMT) (16). Similarly, elevated CHRDL1 expression has been associated with significantly better long-term survival rates in both breast cancer and gastric cancer (17, 18). However, in renal cell carcinoma, CHRDL1 may function as a risk factor (hazard ratio HR=1.193) (19). Currently, the potential role of CHRDL1 in pancreatic cancer remains unreported, and its underlying mechanisms require further investigation.

Our research aimed to discover the role of CHRDL1 in pancreatic cancer cells and investigate the associated mechanisms, which could lead to the discovery of a new target for clinical treatment.

## 2 Materials and methods

### 2.1 Cell line culture and xenograft model establish

The human pancreatic cancer cell lines (PANC-1, SW1990) and normal pancreatic cells (hTERT-HPNE) were purchased from the American Type Culture Collection (ATCC). PANC-1 and hTERT-HPNE cells were cultured in dulbecco’s modified eagle medium (DMEM) (Gibco, Thermo Fisher Scientific, CA, USA) containing 10% fetal bovine serum (FBS, Gibco). SW1990 cells were cultured in L-15 medium containing 10% FBS. The culture media for both cell lines contained 1% v/v penicillin/streptomycin (Beyotime, Jiangsu, China). Five-week-old BALB/c male nude mice were purchased from the Animal Centre of The Chongqing Medical University. The animals were kept in housing with a 12-hour light/dark cycle. To establish the xenograft tumor model, human pancreatic cancer cells (PANC-1 or SW1990, 1×10^6^ cells) suspended in 0.1 mL phosphate-buffered saline (PBS) were injected into 18 nude mice via tail vein. After 9 weeks, the mice were anesthetized with sodium pentobarbital. The lungs were collected, fixed, dehydrated, and embedded in paraffin blocks. Tissue sections were prepared and stained with hematoxylin and eosin (H&E) to confirm tumor metastasis. The number of pulmonary tumor nodules was counted under a dissecting microscope, and the average count was calculated from three lung sections per sample.

All animal experiments were performed in compliance with the Guide for the Care and Use of Laboratory Animals. The protocol was approved by the Chongqing Medical University Animal Care and Use Committee in China.

### 2.2 Lentivirus production and generation of stable cell lines

The EGFP and CHRDL1 genes (OriGene, #RC202635) were subcloned into the lentiviral vector pLV-EF1a-IRES-Ametrine. Subsequently, the constructed vector, along with envelope and packaging plasmids, was transfected into HEK 293T cells to produce lentivirus. Viral supernatants were harvested twice at 48 and 72 hours post-transfection and filtered through a 0.45 μm filter. The constructed lentivirus was transduced into PANC-1 and SW1990 cells. Ametrine signal-based FACS sorting was performed to select positive cells, ultimately establishing PANC-1 and SW1990 stable transduced cell lines. The overexpression level was validated by quantitative reverse transcription polymerase chain reaction (RT-qPCR) and Western blotting (WB).

### 2.3 Cell viability assay

Cell viability was assessed using the CCK-8 assay (Beyotime, Jiangsu, China) following the manufacturer’s protocol. Briefly, stably transfected PANC-1 and SW1990 cells (OE-NC and OE-CHRDL1) were seeded in 96-well plates. After 24 hours, 10 μL of CCK-8 solution was added to each well, followed by incubation at 37°C for 60 minutes. Absorbance at 450 nm was measured using a microplate reader (Thermo Fisher Scientific, Waltham, MA, USA).

### 2.4 Wound-healing assay and cell migration assay

The lentivirus-stably transfected PANC-1 and SW1990 cells (OE-NC or OE-CHRDL1) were seeded in 24-well plates. For the wound healing assay, upon reaching confluent monolayers, cells were treated with BMP4 (2.5 ng/mL, HY-124697, MCE, China) for 1 hour followed by medium replacement. A straight wound was created using a sterile pipette tip, with images captured at 0 and 24 hours post-wounding. Given BMP4’s potent stimulatory effect on migration, the BMP4-treated groups were imaged at 0 and 12 hours instead. Wound closure areas were quantified using ImageJ software.

In the Transwell migration assay, cells were plated in the upper chambers (8 μm pores, Millipore). After 24 hours of incubation, migrated cells on the membrane were fixed with 4% paraformaldehyde and stained with crystal violet. Similarly, for BMP4-treated groups, cells were harvested at the 12-hour time point. Five random fields per group were imaged under microscopy, and the experiment was independently repeated three times. All images were acquired under an optical microscope (Nikon, Tokyo, Japan).

### 2.5 Cell invasion assay

The lentivirus-stably transfected PANC-1 and SW1990 cells (OE-NC or OE-CHRDL1) were seeded into 5 cm serum-free culture dishes pre-coated with 60 μl Matrigel (200 μg/μl, Corning) for 2 hours at room temperature. The lower chambers were filled with culture medium containing 20% fetal bovine serum. After 36 hours of incubation, non-invaded cells on the upper surface were removed with cotton swabs, while invaded cells on the lower membrane were fixed with paraformaldehyde and stained with crystal violet. Five random fields per group were imaged under microscopy, and the experiment was independently repeated three times.

### 2.6 Quantitative real-time PCR

Quantification of CHRDL1 mRNA levels was performed by qPCR with following primers: CHRDL1, forward (5’-CCTGGAACCTTATGGGTTGGT-3’) and reverse (5’- AACATTTGGACATCTGACTCGG-3’); GAPDH, forward (5’- GGAGCGAGATCCCTCCAAAAT-3’) and reverse (5’- GGCTGTTGTCATACTTCTCATGG-3’).

Total RNA was extracted from samples by TRIzol reagent (Invitrogen, Carlsbad, CA, USA), and the concentration and quality of RNA were assessed by a NanoDrop 2000 spectrophotometer (Thermo Fisher Scientific, Waltham, MA, USA). Subsequently, the PrimeScriptTM RT Master Mix (TaKaRa, Tokyo, Japan) was used to synthesis cDNA and qPCR was performed with a SYBR Green qPCR kit. The relative expression levels were calculated using the 2^-ΔΔCt^ method and normalized to GAPDH as reference.

### 2.7 Western blot analysis

Protein samples (50 μg) were isolated from PANC-1 and SW1990 cells by Western and IP cell lysates containing protease inhibitors (Beyotime Biotechnology, Shanghai, China). The protein concentration was quantified by the BCA protein assay kit. The extracted proteins were separated by 10% SDS-PAGE and transferred onto nitrocellulose membranes. After incubation with the appropriate primary antibodies overnight at 4°C, fluorescently labeled goat anti-rabbit IgG (1:15,000, Proteintech, Wuhan, China, 60004-1-Ig, RGAR002) or anti-mouse IgG (1:15,000, Proteintech, Wuhan, China, 60004-1-Ig, RGAM004) was used to detect the primary antibodies. The Odyssey Western Blot Detection System (LI-COR Biotechnology, Lincoln, Nebraska, NE, USA) was employed to visualize the bands and perform image analysis.

The antibodies used for Western blot detection were as follows: p-SMAD 1/5/9 (1:1,1000, Cell Signaling Technology, MA, USA, #9516), SMAD 1/5/9 (1:500, Cell Signaling Technology, MA, USA, #12656), RUNX2 (1:1,1000, Abcam, Cambridge, UK, ab192256), CHRDL1 (1:1,1000, Abcam, Cambridge, UK, ab227473), GAPDH (1:20,000, Proteintech, Wuhan, China, 60004-1-Ig).

### 2.8 Immunohistochemistry staining

Immunohistochemical staining was performed on tumor tissues and adjacent normal tissues from 6 pancreatic cancer patients. Paraffin-embedded tissue sections (4 μm thick) were deparaffinized and dehydrated, followed by blocking of endogenous peroxidase activity with 3% hydrogen peroxide solution. After antigen retrieval, the sections were blocked with 5% bovine serum albumin (BSA) and incubated overnight at 4°C with primary CHRDL1 antibody (1:1,100, Abcam, Cambridge, UK, ab227473), then with secondary antibody (1:100, Proteintech, Wuhan, China, 60004-1-Ig, RGAR002) at 37°C for 1 hour. The sections were developed with DAB (Beyotime, Jiangsu, China) and counterstained with hematoxylin. Images were acquired using a Viewpoint M8 digital slide scanning system (PreciPoint, Freising, Germany).

The ImageJ image analysis system was used for positive scoring: negative (0 points), weakly positive pale yellow (1 point), moderately positive brownish yellow (2 points), and strongly positive brown (3 points). The weakly, moderately, and strongly positive areas, tissue area, and integrated optical density (IOD) values of positive signals were calculated. The histochemical score (H-Score) was determined using the formula: H-Score = ∑(pi×i) = (percentage of weakly positive area × 1) + (percentage of moderately positive area × 2) + (percentage of strongly positive area × 3), where pi represents the percentage of positive signal area and i represents the positive intensity grade (20).

### 2.9 Histological analysis

The lung specimens were fixed in 4% paraformaldehyde at 4°C for 24 hours, followed by dehydration through a graded ethanol series and clearing in xylene before paraffin embedding. The paraffin-embedded samples were sectioned at 4 μm thickness using a microtome. Tissue sections were stained with hematoxylin for 5–10 minutes and counterstained with eosin for 1 minute, then mounted for histological examination. Digital images were acquired using a Viewpoint M8 digital slide scanning system (Precipoint, Freising, Germany) and tumor areas were quantified using ImageJ software.

### 2.10 Statistical analysis

Data were presented as the mean ± SEM. All statistical analysis was performed using SPSS 26.0 (SPSS, Inc., Chicago, IL, USA). Statistical significance was determined by Student’s t-test or one-way ANOVA, followed by Sidak’s multiple comparisons test for multi-group. All tests were two-sided with a *P*-value of 0.05 used to determine statistical significance.

## 3 Results

### 3.1 Decreased expression of CHRDL1 in pancreatic cancer

**Figure 1:**
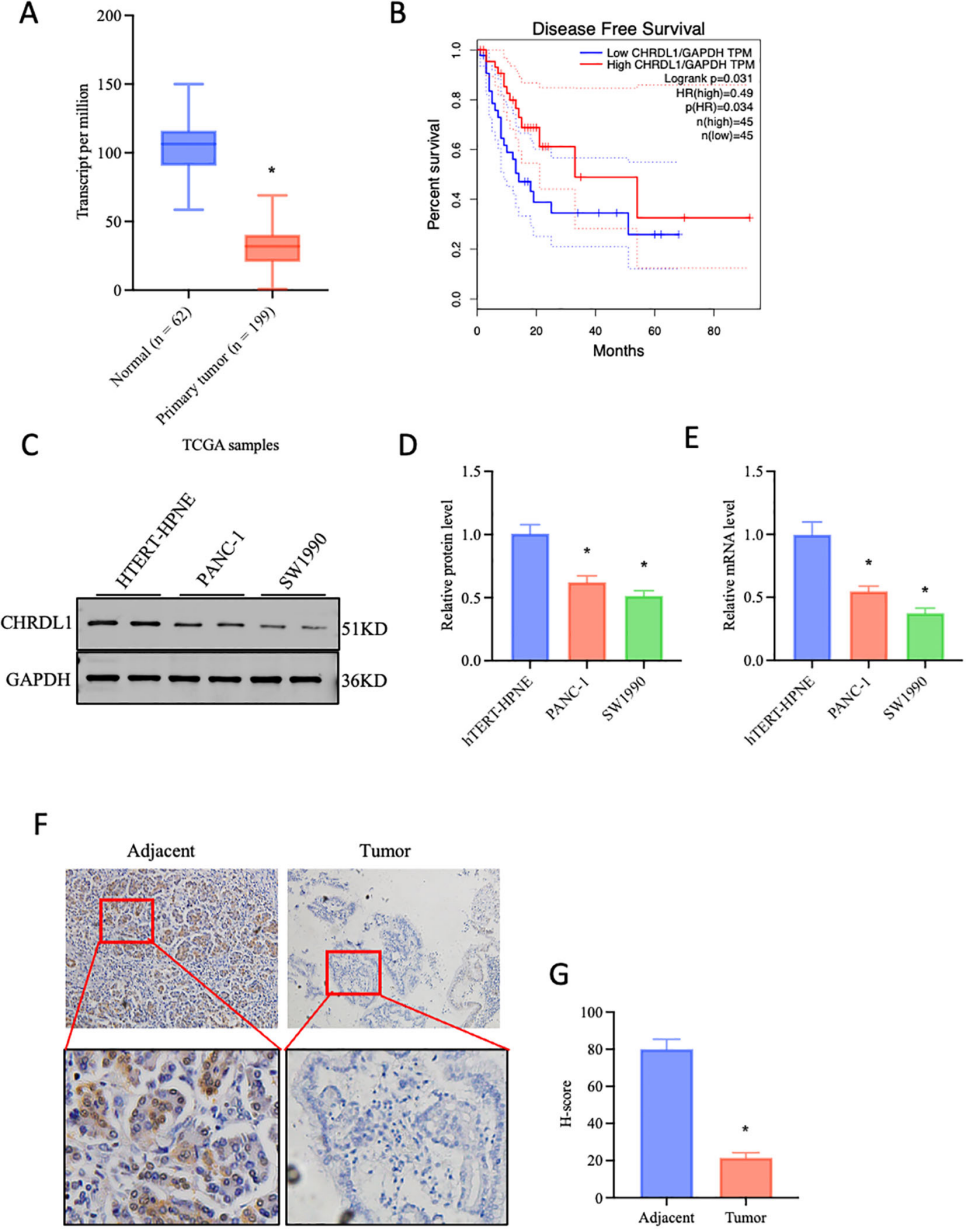
Decreased expression of CHRDL1 in pancreatic cancer. **(A)** Data-based prediction of CHRDL1 expression in pancreatic cancer cells, two-tailed Student’s t-test. **(B)** Association between CHRDL1 and disease-free survival (DFS). **(C-E)** The efficiency of CHRDL1 overexpression in the pancreatic cancer cell lines PANC-1 and SW1990 was verified using quantitative real-time PCR (RT-qPCR) and Western blot analysis (n = 6, *P < 0.05 vs. hTERT-HPNE, one-way ANOVA followed by Sidak’s multiple comparisons test for multi-group). **(F, G)** IHC analysis of CHRDL1 in tumor and adjacent tissues (n = 6, *P < 0.05 vs. adjacent, two-tailed Student’s t-test).

Data from The Cancer Genome Atlas (TCGA) was utilized to explore the correlation between CHRDL1 expression and pancreatic cancer development. It was found that CHRDL1 was significantly downregulated in pancreatic cancer tumors compared to normal tissues (**Figure 1A**). Moreover, higher CHRDL1 expression was positively associated with improved overall survival (**Figure 1B**), suggesting a potential protective role in pancreatic cancer. Based on these observations, we hypothesized that CHRDL1 may play a regulatory role in pancreatic cancer progression. To validate this, we examined CHRDL1 expression in pancreatic cancer cell lines (PANC-1, SW1990). Both protein and mRNA levels of CHRDL1 were markedly reduced in PANC-1 and SW1990 cells compared to normal pancreatic cells (**Figures 1C–E**). Further supporting this, immunohistochemical (IHC) staining of pancreatic tissues from a mouse model demonstrated that CHRDL1 was diffusely localized in the cytoplasm of normal pancreatic acinar cells. However, its expression was significantly diminished in pancreatic tumor tissues (**Figures 1F, G**). These findings suggest that CHRDL1 is downregulated in pancreatic cancer tissues and serves as a favorable prognostic factor in pancreatic cancer.

### 3.2 Overexpression of CHRDL1 inhibits malignant biological behaviors of PANC-1 and SW1990 cells

**Figure 2:**
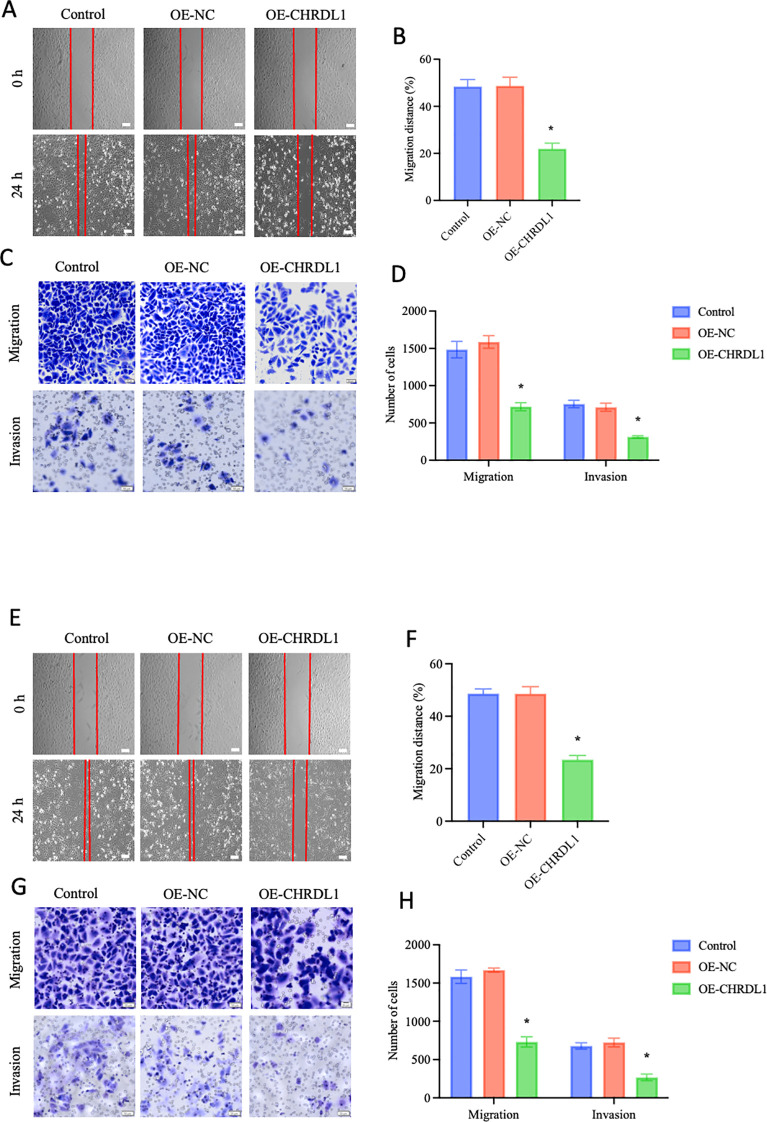
Overexpression of CHRDL1 inhibits malignant biological behaviors of PANC-1 and SW1990 cells. **(A, B)** Representative images of wound healing at 0 and 24 h in CHRDL1 transduced PANC-1 cells, along with statistical results of migration distance percentages for each group (Scale bars: 100 μm). **(C, D)** Migration and invasion assays of transfected PANC-1 cells, showing representative images and corresponding statistical analysis for each group (scale bars, upper: 50 μm, lower: 20 μm). **(E, F)** Representative images of wound healing at 0 and 24 h in CHRDL1 transduced SW1990 cells, along with statistical results of migration distance percentages for each group (Scale bars: 100 μm). **(G, H)** Migration and invasion assays of transfected SW1990 cells, showing representative images and corresponding statistical analysis for each group (scale bars, upper: 50 μm, lower: 20 μm). n = 6 in each group, *P < 0.05 vs. control, one-way ANOVA followed by Sidak’s multiple comparisons test for multi-group.

To further investigate whether CHRDL1 affects the progression of pancreatic cancer, CHRDL1 was overexpressed in PANC-1 and SW1990 cells. RT-qPCR and Western blot were used to assess the efficiency of CHRDL1 overexpression (**Supplementary Figures 1A–F**). Previous studies have identified CHRDL1 as a negative regulator that primarily suppresses breast cancer cell migration and invasion (17). To examine whether CHRDL1 exerts similar phenotypic effects in pancreatic cancer, we conducted functional assays using PANC-1 and SW1990 cell lines. Wound healing and migration assays performed on PANC-1 cells revealed that CHRDL1 overexpression significantly attenuated cellular migration capacity. Furthermore, adhesion experiments demonstrated that elevated CHRDL1 expression inhibited PANC-1 cell adhesion (**Figures 2A–D**). Proliferation assays showed no significant effect of CHRDL1 overexpression on PANC-1 cell growth (**Supplementary Figure 1G**). parallel experiments in SW1990 cells yielded consistent results, with CHRDL1 overexpression similarly suppressing migratory and adhesive capabilities while exhibiting no measurable impact on proliferation rates (**Figures 2E–H**, **Supplementary Figure 1H**). These findings collectively demonstrate that CHRDL1 functions as a metastasis suppressor in pancreatic cancer through specific inhibition of migration and adhesion processes, without influencing proliferative activity, thereby extending its known tumor-suppressive role beyond breast cancer to pancreatic malignancies.

### 3.3 Overexpression of CHRDL1 inhibits BMP4-induced SMAD phosphorylation in pancreatic cancer cells

**Figure 3:**
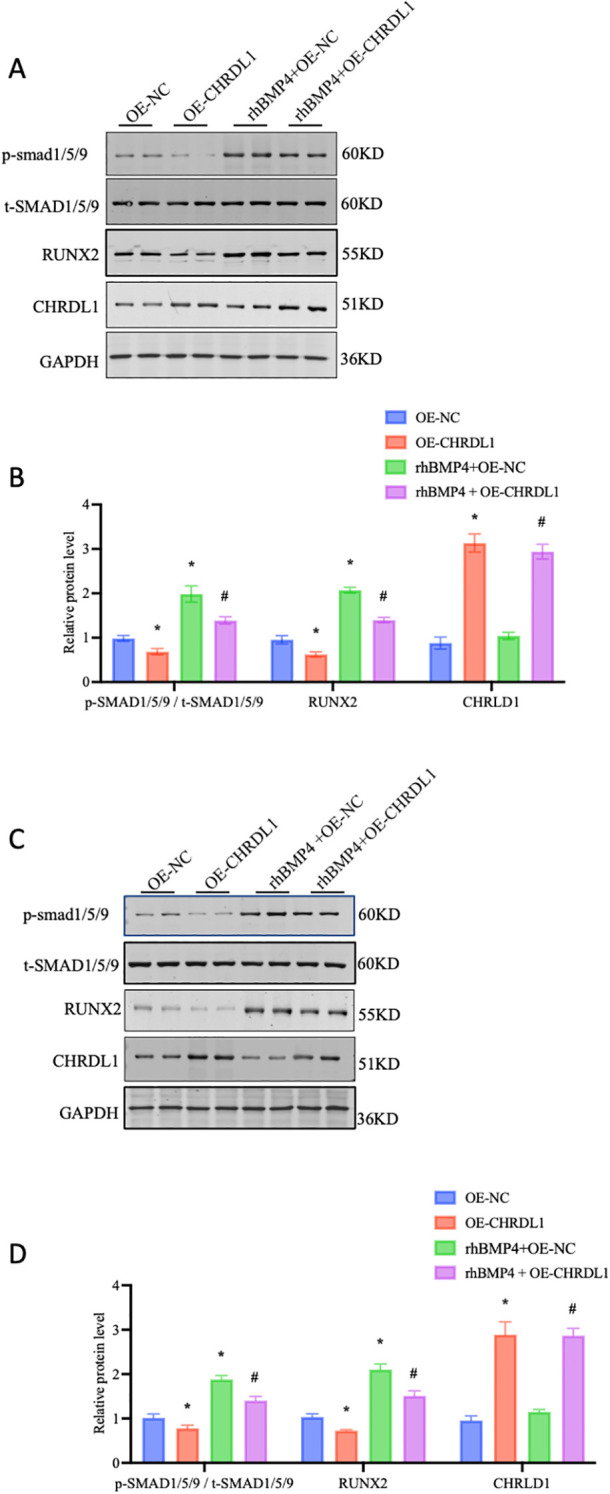
Overexpression of CHRDL1 inhibits BMP4-Induced SMAD phosphorylation in pancreatic cancer cells. **(A, B)** p-SMAD 1/5/9, t-SMAD 1/5/9, RUNX2 and CHRDL1 protein levels in PANC-1 cells were detected using Western blots. The immunoblots were calculated by densitometric analysis using GAPDH as the internal reference. **(C, D)** p-SMAD 1/5/9, t-SMAD 1/5/9, RUNX2 and CHRDL1 protein levels in SW1990 cells were detected using Western blots. The immunoblots were calculated by densitometric analysis using GAPDH as the internal reference. n = 6 in each group, *P < 0.05 vs. OE-NC, **^#^** *P* < 0.05 rhBMP4+OE-NC, one-way ANOVA followed by Sidak’s multiple comparisons test for multi-group.

CHRDL1 is a BMP signaling antagonist, exhibiting high affinity for BMP4 in particular (21). CHRDL1 has been demonstrated to exert its effects in various breast cancer cell lines by antagonizing the BMP/SMAD signaling pathway (17). Therefore, we attempted to use recombinant BMP4 protein (2.5 ng/ml) to verify its effects in the progression of pancreatic cancer cells. The PANC-1 and SW1990 cell lines were overexpressed with CHRDL1 lentivirus, followed by treatment with recombinant BMP4 protein for 1 hour. In the PANC-1 cell line, CHRDL1 overexpression significantly increased protein levels compared to the control group, confirming successful overexpression at the protein level. Subsequent experiments revealed that CHRDL1 overexpression led to a moderate reduction in RUNX2 protein expression and SMAD1/5/9 phosphorylation levels. While recombinant BMP4 protein treatment markedly enhanced both RUNX2 expression and SMAD1/5/9 phosphorylation, these BMP4-induced effects were partially reversed in the context of CHRDL1 overexpression (**Figures 3A, B**). Parallel experiments conducted in SW1990 cells yielded consistent results, demonstrating similar regulatory patterns (**Figures 3C, D**). These findings collectively indicate that CHRDL1 functions as an antagonist capable of inhibiting the BMP4/SMAD signaling pathway across various pancreatic cancer cell lines.

### 3.4 CHRDL1 attenuates BMP4-induced malignant biological behaviors of pancreatic cancer cells

**Figure 4:**
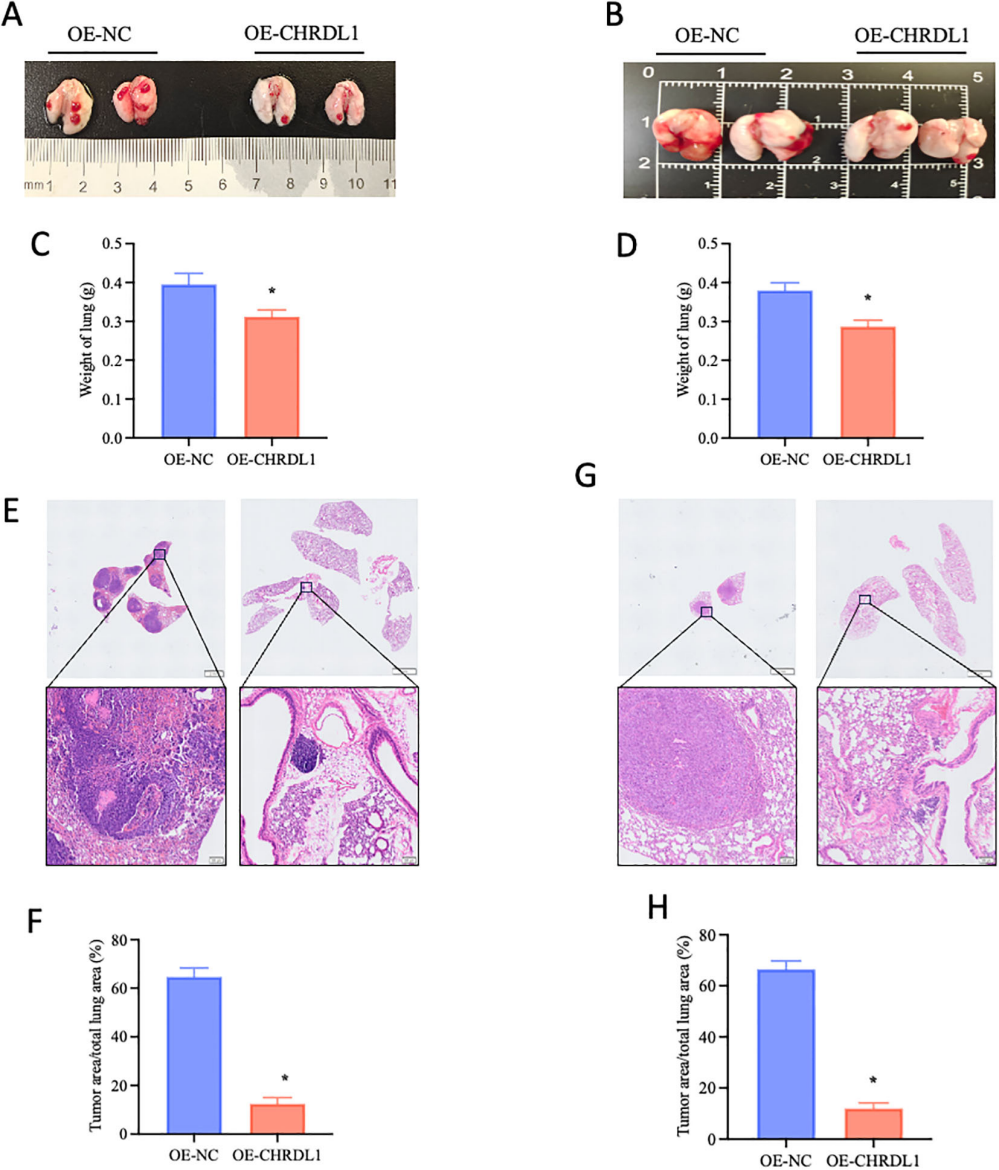
CHRDL1 attenuates BMP4-induced malignant biological behaviors of pancreatic cancer cells. **(A, B)** Representative wound healing images of CHRDL1-transduced PANC-1 cells treated with rhBMP4 at 0 and 12 hours, along with statistical results of migration distance percentages for each group (Scale bars: 100 μm). **(C, D)** Migration and invasion assays of transfected PANC-1 cells treated with rhBMP4, showing representative images and corresponding statistical analysis for each group (scale bars, upper: 50 μm, lower: 20 μm). **(E, F)** Representative wound healing images of CHRDL1-transduced SW1990 cells treated with rhBMP4 at 0 and 12 hours, along with statistical results of migration distance percentages for each group (Scale bars: 100 μm). **(G, H)** Migration and invasion assays of transfected SW1990 cells treated with rhBMP4, showing representative images and corresponding statistical analysis for each group (scale bars, upper: 50 μm, lower: 20 μm).n = 6 in each group, *P < 0.05 vs. OE-NC, one-way ANOVA followed by Sidak’s multiple comparisons test for multi-group.

The BMP/SMAD signaling pathway is always regarded as a promoter of malignant behaviors in pancreatic cancer cells (22). Gordon KJ et al. found that recombinant BMP enhances the migration and adhesion abilities of pancreatic cancer cells (23). Thus, we aimed to determine whether CHRDL1, through its antagonist properties, could reverse this effect. Using our established PANC-1 cell line with stable CHRDL1 overexpression, we investigated its biological function by treating cells with recombinant BMP4 for 1 hour and observing effects after 12 hours. Compared to control groups, BMP4 treatment significantly enhanced cellular migration and adhesion capabilities, effects that were partially reversed by CHRDL1 overexpression (**Figures 4A–D**). Parallel experiments in SW1990 cells confirmed this antagonistic effect of CHRDL1 against BMP4 (**Figures 4E–H**). These findings collectively demonstrate that CHRDL1 functions as a BMP4 antagonist capable of suppressing malignant behaviors in pancreatic cancer cells through modulation of the BMP4/SMAD signaling pathway.

### 3.5 CHRDL1 inhibits pancreatic cancer metastasis *in vivo*

**Figure 5:**
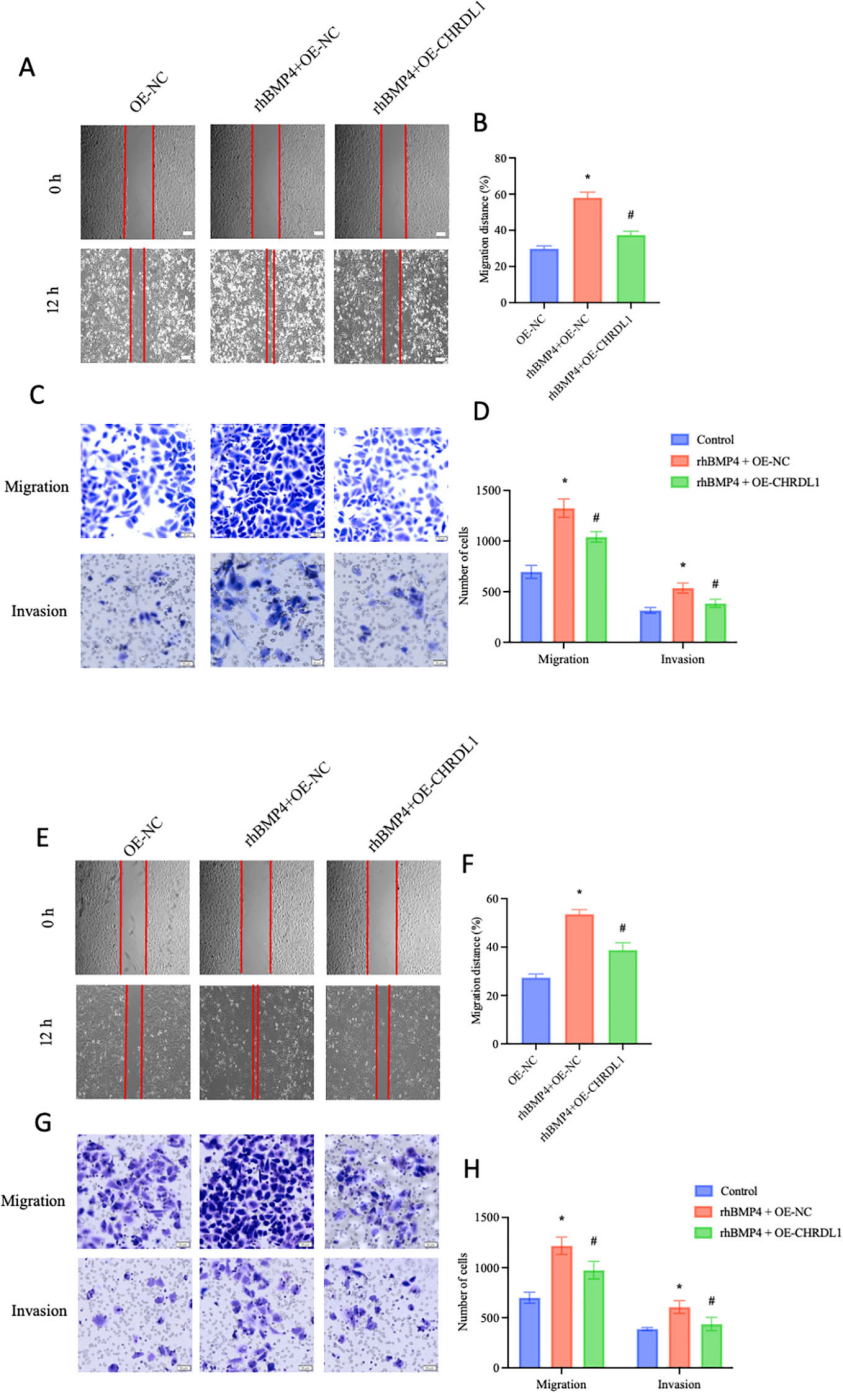
CHRDL1 inhibits pancreatic cancer metastasis *in vivo.* **(A, B)** Representative lung images of BALB/c nude mice at 9 weeks after tail vein injection with OE-NC/OE-CHRDL1 stably transfected PANC-1 cells (left) or SW1990 cells (right). **(C, D)** Lung weights of BALB/c nude mice at 9 weeks after tail vein injection with OE-NC/OE-CHRDL1 stably transfected PANC-1 cells (left) or SW1990 cells (right). **(E-H)** H&E-stained lung sections and corresponding quantitative analysis of tumor area/total lung area from mice injected with OE-NC/OE-CHRDL1 stably transfected PANC-1 cells (left) or SW1990 cells (right). Scale bars, upper: 2 mm, lower: 100 μm. n = 6 in each group, *P < 0.05 vs. OE-NC, two-tailed Student’s t-test.

Given that the effects of CHRDL1 primarily targeted tumor migration and adhesion rather than proliferation, we established a xenograft model to evaluate its impact on *in vivo* metastasis. Using stably transfected PANC-1 and SW1990 cell lines (OE-NC or OE-CHRDL1), we intravenously injected these cells via the tail vein into 4-week-old male BALB/c nude mice to assess lung colonization capacity. Specimens collected after 9 weeks revealed that compared to the OE-NC group, both OE-CHRDL1 PANC-1 and SW1990 cells exhibited significantly reduced metastatic potential (**Figures 5A, B**). Lung weight measurements showed marked reduction in mice injected with OE-CHRDL1 cells versus OE-NC controls (**Figures 5C, D**). Histopathological examination (H&E staining) of harvested lung tissues demonstrated fewer tumor infiltrates and lower tumor-to-lung area ratios in OE-CHRDL1 groups, whereas OE-NC groups displayed extensive pulmonary tumor colonies (**Figures 5E–H**). These results conclusively demonstrate that CHRDL1 overexpression suppresses tumor metastasis and pulmonary colonization *in vivo*.

## 4 Discussion

With the advent of an aging society, the global incidence of pancreatic cancer has increased 2.3 times from 1990 to 2017, affecting approximately 441,000 patients (3). Besides genetic factors, risk factors such as smoking, heavy alcohol drinking, high body mass index, and diabetes continue to play significant roles in the risk of pancreatic cancer, exacerbating the global burden of the disease (24). Our study provided evidence that CHRDL1 inhibited the progression of pancreatic cancer in a mouse xenograft model. Overexpression of CHRDL1 inhibited the migration and adhesion of pancreatic cancer cells (PANC-1 and SW1990) and significantly reduced the tumor weight and size in mice. Mechanistically, CHRDL1 exerts its tumor-suppressive effects by antagonizing the BMP4/SMAD signaling pathway.

CHRDL1 is a secreted glycoprotein and a BMP antagonist associated with various cancers (25). The expression of CHRDL1 is downregulated in gastric cancer, thyroid cancer, lung cancer, malignant melanoma, and breast cancer (17, 18, 26–28). In our study, we found that CHRDL1 expression was lower in pancreatic cancer tissues compared to adjacent tissues using RT-qPCR, Western blotting, and immunohistochemistry. Meanwhile, lower CHRDL1 expression was associated with poorer prognosis in pancreatic cancer patients. Analysis of the TCGA database indicated that CHRDL1 was downregulated in pancreatic cancer cells. Additionally, we validated the lower expression of CHRDL1 in PANC-1 and SW1990 cells when compared with normal pancreatic cells. This suggests that CHRDL1 is lowly expressed in pancreatic cancer and is correlated with poor prognosis in pancreatic cancer patients.

We further investigated whether CHRDL1 plays a role in pancreatic cancer progression. Tumor metastasis *in vivo* primarily involves five key steps: local invasion, intravasation, circulatory survival, extravasation, and colonization (29). Our study demonstrated that CHRDL1 overexpression suppresses the migration and adhesion of pancreatic cancer cells (PANC-1 and SW1990) without significantly affecting their proliferation, which aligns with the reported effects of CHRDL1 in breast cancer and melanoma cells (17, 28). However, this contrasts with findings in gastric cancer (18), where CHRDL1 was shown to inhibit proliferation, suggesting tissue-specific differences in CHRDL1-mediated signaling. To further examine the functional impact of CHRDL1 in pancreatic cancer metastasis, we intravenously injected CHRDL1-transfected tumor cells into nude mice and assessed their metastatic potential. Overexpression of CHRDL1 in both PANC-1 and SW1990 cells significantly reduced the number of lung tumor nodules and their relative occupied area. These results indicate that CHRDL1 overexpression effectively suppresses the metastatic spread of pancreatic cancer cells *in vivo.*

BMPs have the ability to induce tumorigenesis and regulate cancer progression at various stages (30). During early tumor development, BMP signaling may promote epithelial-mesenchymal transition (EMT) through SMAD1/5/9 phosphorylation, while in advanced stages, it may potentially suppress excessive proliferation of metastatic foci (31, 32). CHRDL1, as a BMP antagonist, possesses repeated cysteine-rich (CR) domains that prevents binding of BMP ligands to their receptors, thereby blocking the activation of BMP signaling (10). Studies have shown that CHRDL1 promotes the proliferation and migration of gastric cancer cells by antagonizing BMPR II (18). Besides, high levels of CHRDL1 inhibited BMP4-induced Smad1/5/8 phosphorylation, thereby suppressing tumor growth (17). We also aimed to verify whether CHRDL1 regulated the malignant behaviors of pancreatic cancer cells through the BMP/SMAD pathway. We found that overexpression of CHRDL1 significantly decreased the protein levels of BMP and p-SMAD1/5/9, which was consistent with previous findings. Notably, SMAD4 - a tumor suppressor gene frequently deleted in pancreatic cancer - may form a feedback loop with BMP4 regulation (33). The enhanced dependence on the BMP/SMAD1/5/9 pathway in SMAD4-deficient contexts could potentially explain why CHRDL1 exhibits more pronounced metastasis-suppressing effects in pancreatic cancer (34). Subsequently, we added recombinant BMP4 protein to CHRDL1-overexpressing PANC-1 and SW1990 cells and observed impaired cell migration and adhesion abilities. This indicates that CHRDL1 exerts its effects on inhibiting the malignant behaviors of pancreatic cancer cells by relying on the inhibition of the BMP/SMAD pathway.

## 5 Conclusion

**Figure 6:**
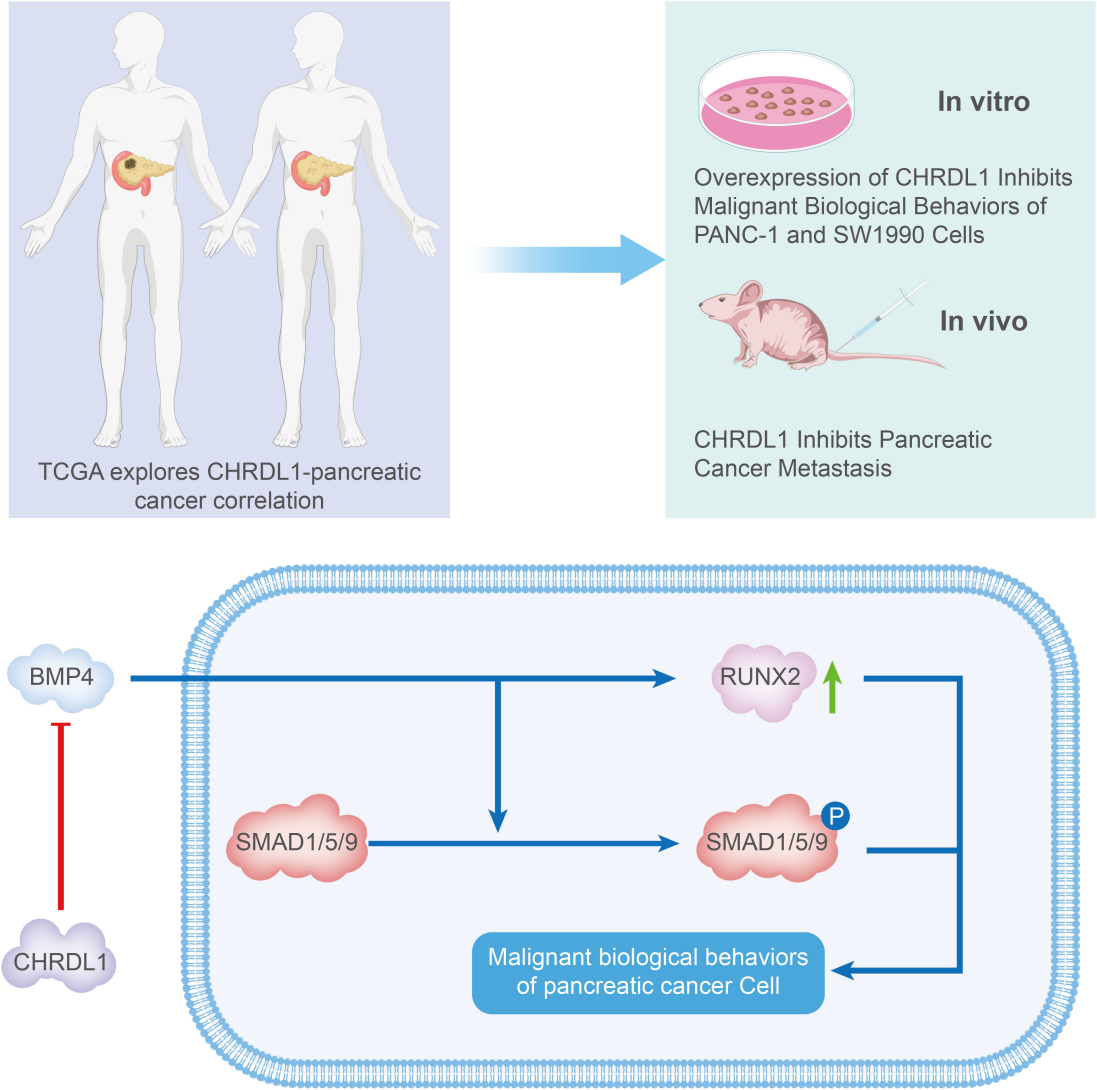
Schematic illustration of the mechanism whereby CHRDL1 regulates pancreatic cancer. In general, CHRDL1 functions as a BMP antagonist that desensitizes pancreatic cancer cells to BMP-4, thereby inhibiting BMP-mediated SMAD1/5/9 signaling activation. Ultimately, CHRDL1 suppresses malignant behaviors of pancreatic cancer cells and impedes tumor progression *in vivo*.

In summary, our study confirmed that CHRDL1 exerts an inhibitory effect on pancreatic cancer both in vitro and in vivo, with its mechanism illustrated in **Figure 6**. As a BMP antagonist, CHRDL1 desensitizes pancreatic cancer cells to BMP-4, thereby inhibiting BMP-mediated SMAD1/5/9 signaling. Ultimately, CHRDL1 suppressed the malignant behaviors of pancreatic cancer cells and the progression of pancreatic cancer *in vivo*. By inhibiting the BMP/SMAD signaling pathway, CHRDL1 might elucidate crucial mechanisms in pancreatic cancer and represented a potential therapeutic target for the treatment of this disease.

## Data Availability

The original contributions presented in the study are included in the article/**Supplementary Material**. Further inquiries can be directed to the corresponding author.

## Supplementary material

Supplementary Figure 1
**(A-D)** Western blot was performed to detect and quantitatively analyze CHRDL1 protein expression in the stably transduced PANC-1 cells (left) or SW1990 cells (right). The immunoblots were calculated by densitometric analysis using GAPDH as the internal reference. **(E, F)** Relative expression level of CHRDL1 mRNA after lentiviral transfection and establishment of stable PANC-1 cells (left) or SW1990 cells (right). **(G, H)** No obvious effect of CHRDL1 on cell viability of PANC-1 cells (left) or SW1990 cells (right), indicated by CCK-8 assay. n = 6 in each group, **P* < 0.05 vs. OE-NC, one-way ANOVA followed by Sidak’s multiple comparisons test for multi-group.
