# Correction: Chorordin-like 1 inhibits pancreatic cancer cell migration and invasion: involvement of the BMP4/SMAD pathway

**DOI:** 10.3389/fonc.2025.1705973

**Published:** 2025-10-01

**Authors:** Wei Li, Yalan Zhong, Yuqiao Song, Hongmei Wang, Zheng Jiang

**Affiliations:** ^1^ Department of Gastroenterology, The First Affiliated Hospital of Chongqing Medical University, Chongqing, China; ^2^ The Second People’s Hospital of Yubei District, Chongqing, China

**Keywords:** pancreatic cancer, CHRDL1, BMP4/SMAD, migration, invasion

Authors “Yalan Zhong, Yuqiao Song, and Hongmei Wang” were erroneously assigned to affiliation “Department of Gastroenterology, The First Affiliated Hospital of Chongqing Medical University, Chongqing, China”. This affiliation has now been removed for authors “Yalan Zhong, Yuqiao Song, and Hongmei Wang”.

Author “Zheng Jiang” was erroneously assigned to affiliation “Department of Gastroenterology, the Second People’s Hospital of Yubei District, Chongqing, China”. This affiliation has now been removed for author “Zheng Jiang”.

Affiliation “The Second People’s Hospital of Yubei District, Chongqing, China” was erroneously given as “Department of Gastroenterology, the Second People’s Hospital of Yubei District, Chongqing, China”.

The original version of this article has been updated.

