# Supplementary figures and images for: Chorordin-like 1 inhibits pancreatic cancer cell migration and invasion: involvement of the BMP4/SMAD pathway
Source: Front Oncol. 2025 Aug 5;15:1633464. doi: 10.3389/fonc.2025.1633464 (PMC12361159; doi:10.3389/fonc.2025.1633464)

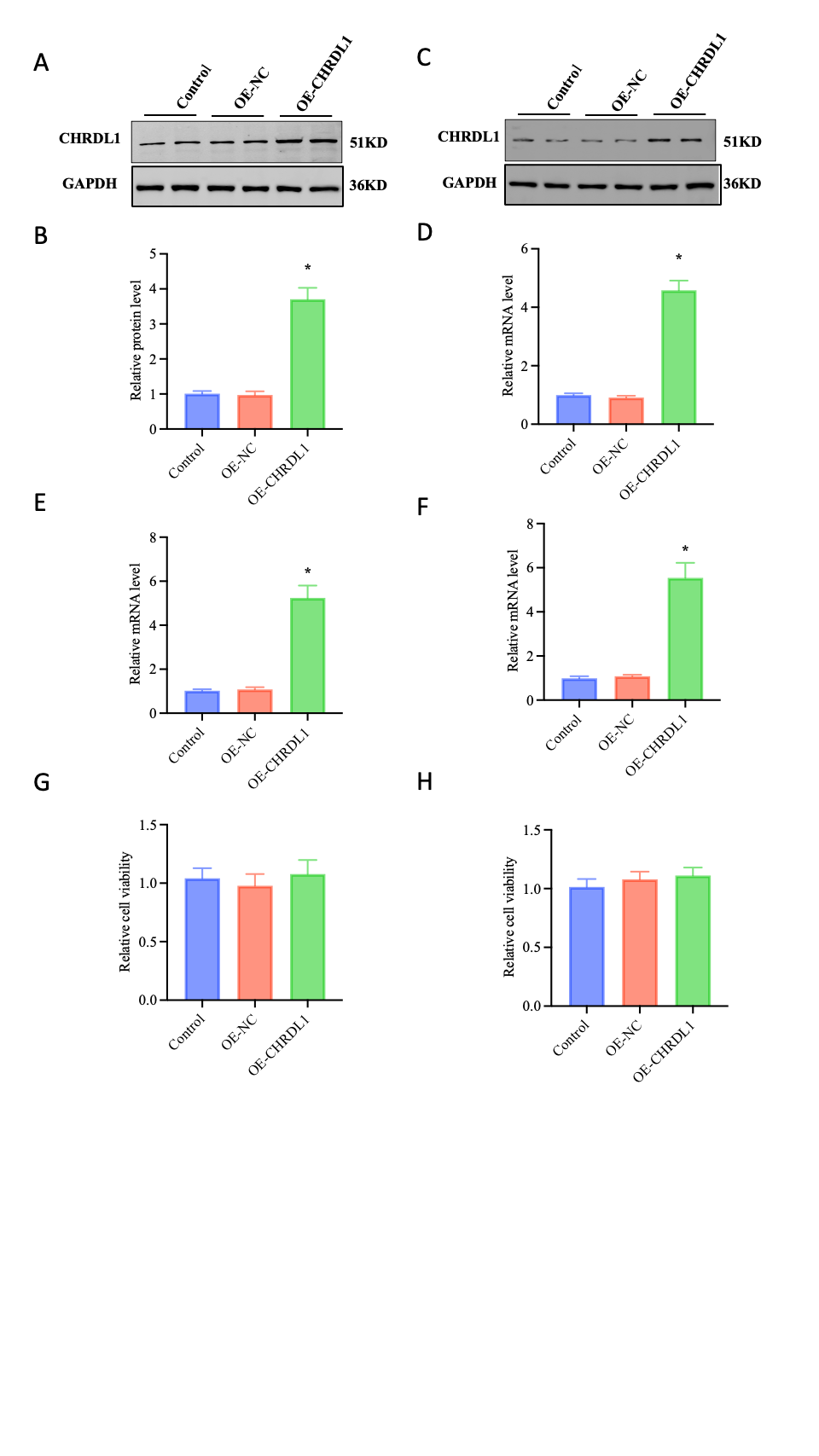

Supplement: Supplementary Figure 1 — (A-D) Western blot was performed to detect and quantitatively analyze CHRDL1 protein expression in the stably transduced PANC-1 cells (left) or SW1990 cells (right). The immunoblots were calculated by densitometric analysis using GAPDH as the internal reference. (E, F) Relative expression level of CHRDL1 mRNA after lentiviral transfection and establishment of stable PANC-1 cells (left) or SW1990 cells (right). (G, H) No obvious effect of CHRDL1 on cell viability of PANC-1 cells (left) or SW1990 cells (right), indicated by CCK-8 assay. n = 6 in each group, *P < 0.05 vs. OE-NC, one-way ANOVA followed by Sidak’s multiple comparisons test for multi-group. [file Image1.tiff]

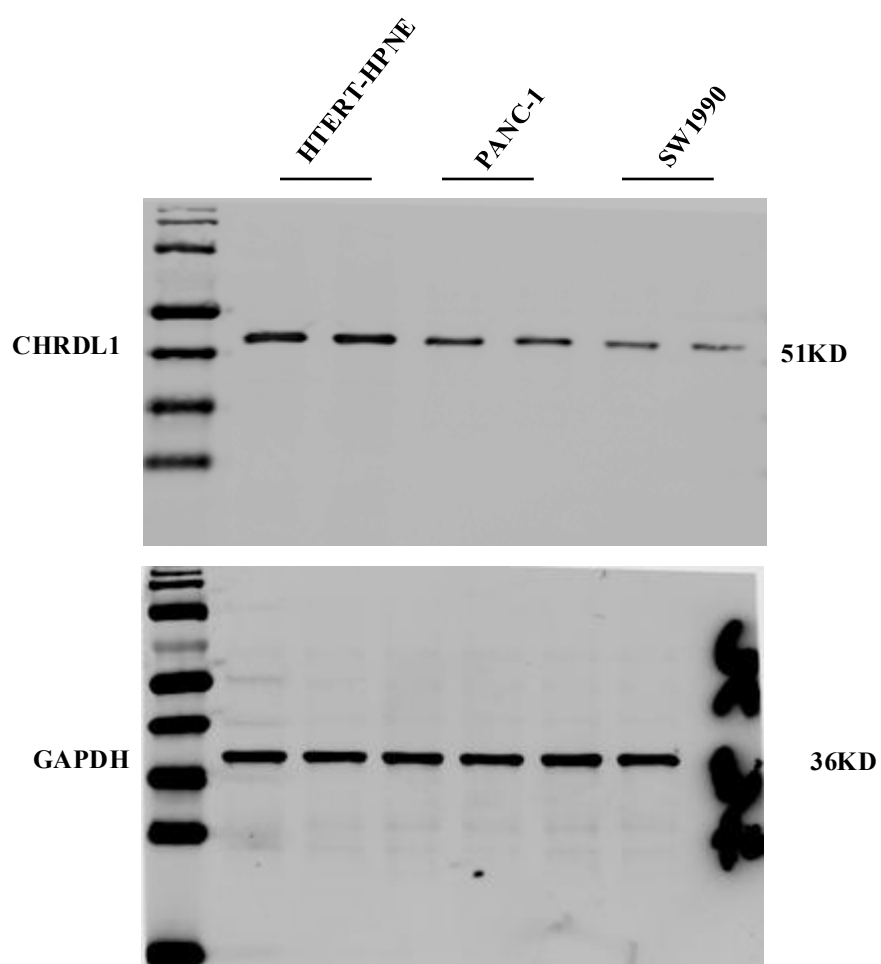

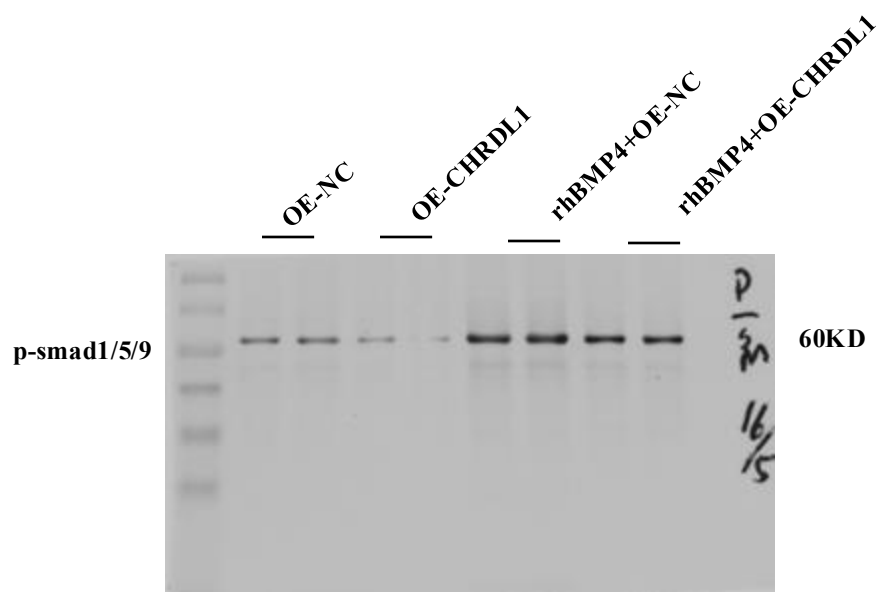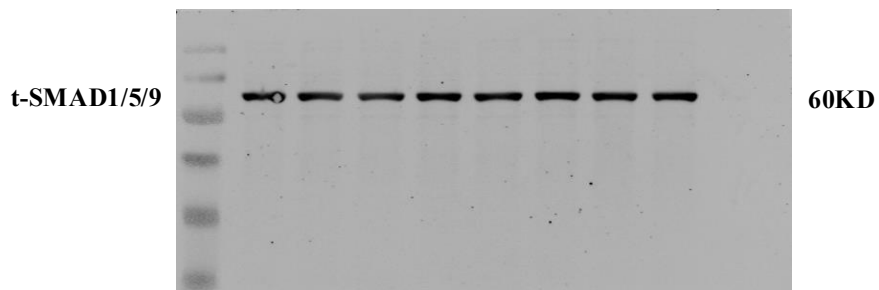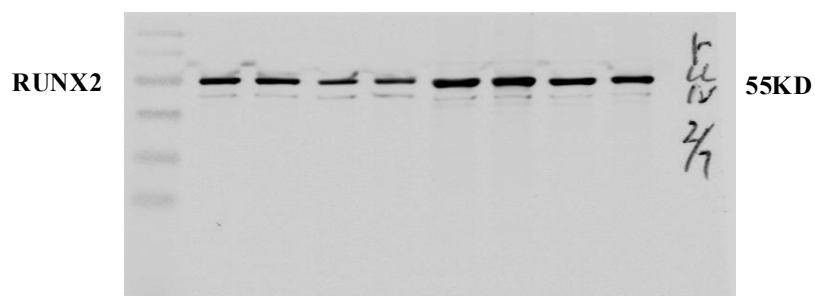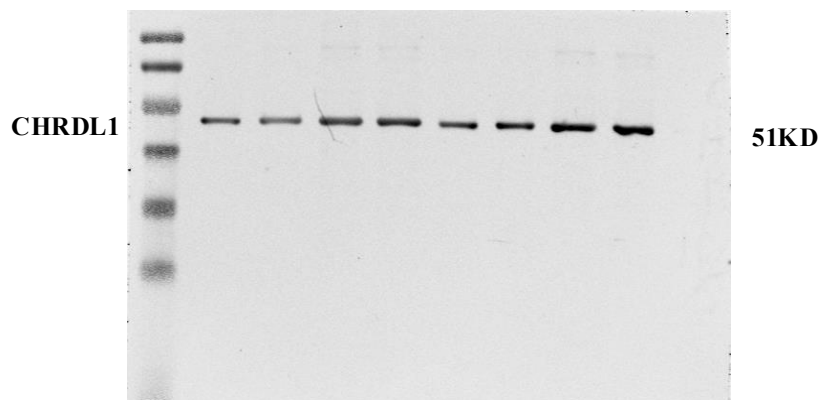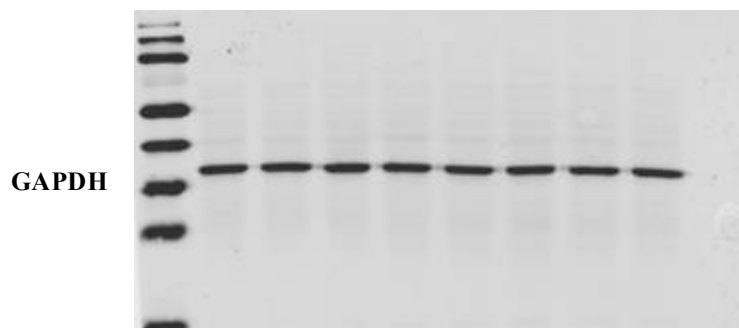

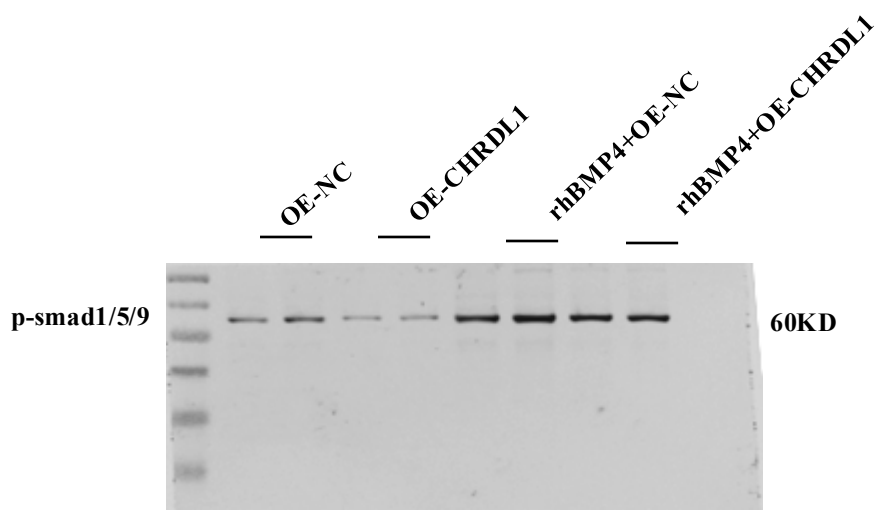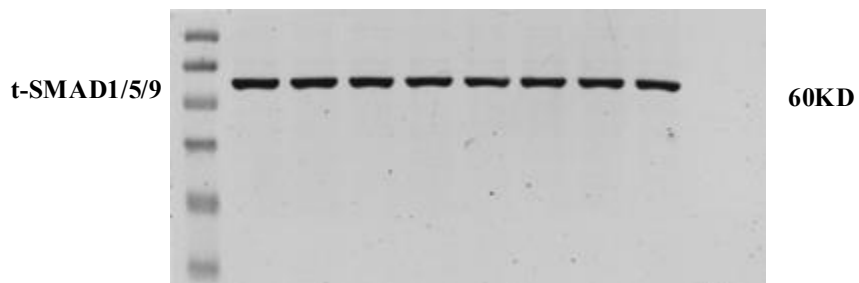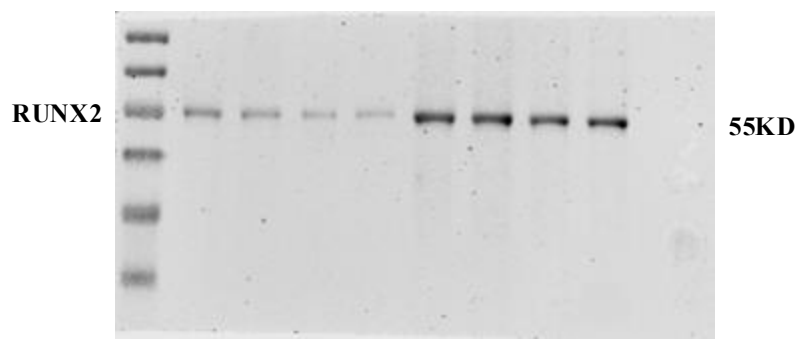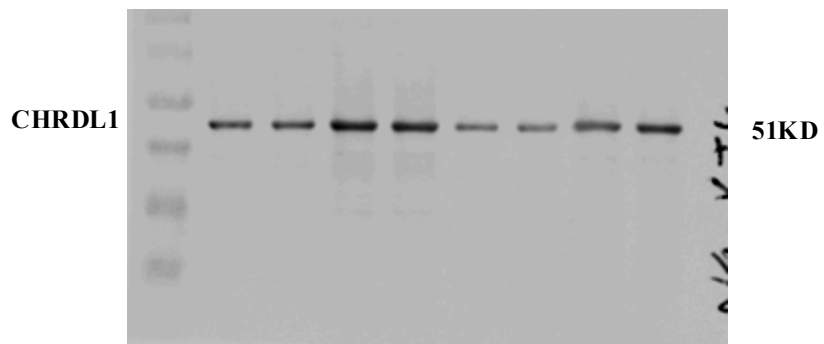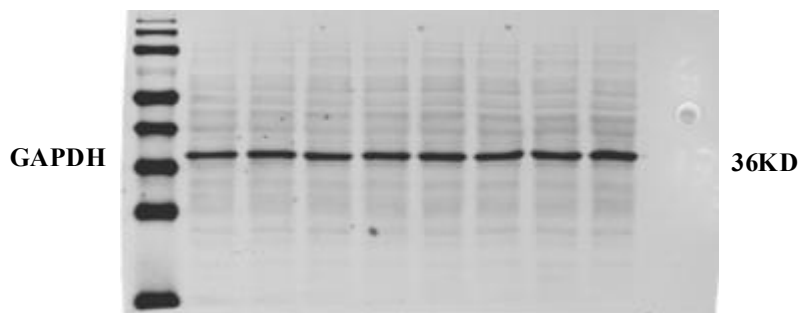

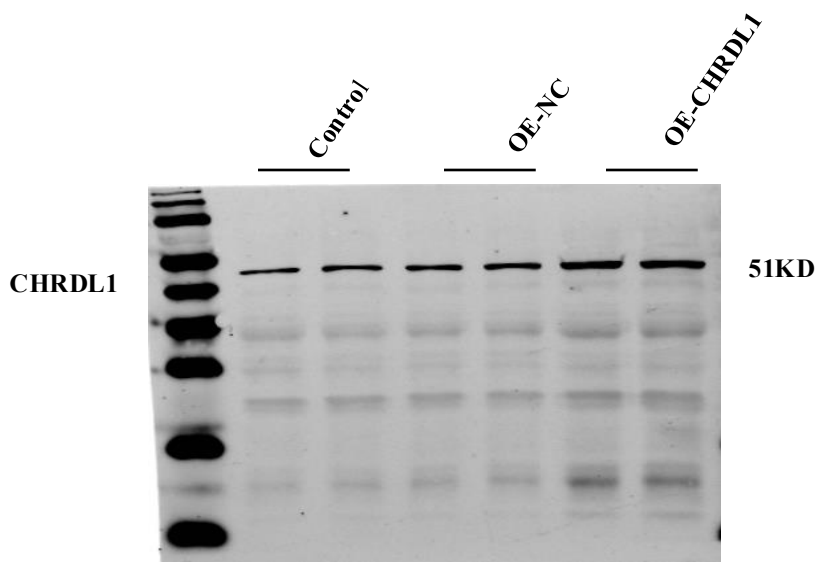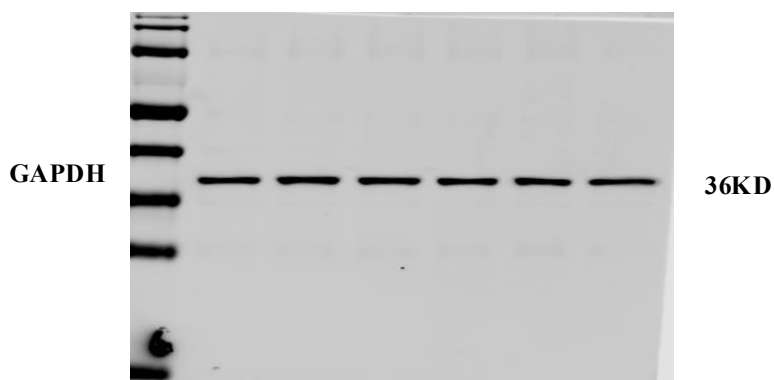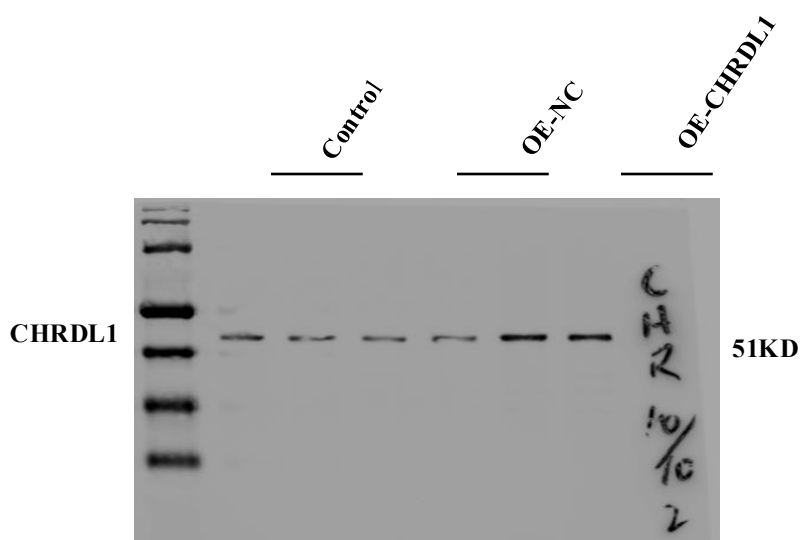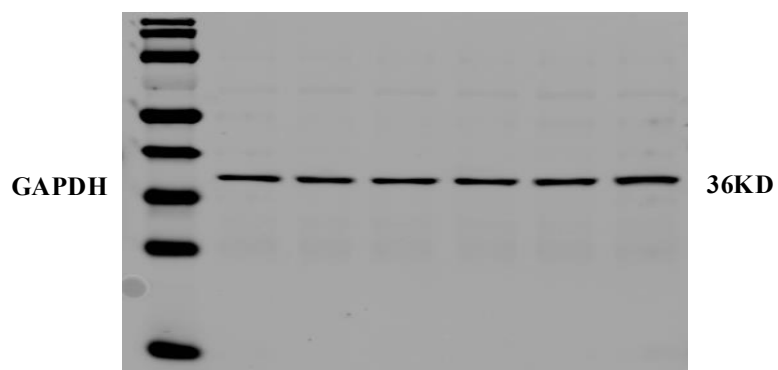

Supplement: Supplementary file 2 [file DataSheet1.pdf]
